# CT-based radiogenomic analysis dissects intratumor heterogeneity and predicts prognosis of colorectal cancer: a multi-institutional retrospective study

**DOI:** 10.1186/s12967-022-03788-8

**Published:** 2022-12-08

**Authors:** Min-Er Zhong, Xin Duan, Ma-yi-di-li Ni-jia-ti, Haoning Qi, Dongwei Xu, Du Cai, Chenghang Li, Zeping Huang, Qiqi Zhu, Feng Gao, Xiaojian Wu

**Affiliations:** 1 Department of Colorectal Surgery, The Sixth Affiliated Hospital, Sun Yat-Sen University, Guangzhou, 510655 China; 2 Department of Gastrointestinal Surgery, Department of General Surgery, Guangdong Provincial People’s Hospital, Guangdong Academy of Medical Sciences, Guangzhou, China; 3 Guangdong Provincial Key Laboratory of Colorectal and Pelvic Floor Diseases, The Sixth Affiliated Hospital, Sun Yat-Sen University, Guangzhou, China; 4 School of Biomedical Engineering, Shenzhen Campus of Sun Yat-sen University, Shenzhen, China; 5 Department of Radiology, The First People’s Hospital of Kashi Prefecture, Kashi, Xinjiang China; 6 Department of Colorectal Surgery, Ningbo Medical Center Lihuili Hospital, Ningbo, China; 7 Shanghai Artificial Intelligence Laboratory, Shanghai, China

**Keywords:** Colorectal cancer, Radiogenomic, Intratumor heterogeneity, Prognosis, Signature

## Abstract

**Background:**

This study aimed to develop a radiogenomic prognostic prediction model for colorectal cancer (CRC) by investigating the biological and clinical relevance of intratumoural heterogeneity.

**Methods:**

This retrospective multi-cohort study was conducted in three steps. First, we identified genomic subclones using unsupervised deconvolution analysis. Second, we established radiogenomic signatures to link radiomic features with prognostic subclone compositions in an independent radiogenomic dataset containing matched imaging and gene expression data. Finally, the prognostic value of the identified radiogenomic signatures was validated using two testing datasets containing imaging and survival information collected from separate medical centres.

**Results:**

This multi-institutional retrospective study included 1601 patients (714 females and 887 males; mean age, 65 years ± 14 [standard deviation]) with CRC from 5 datasets. Molecular heterogeneity was identified using unsupervised deconvolution analysis of gene expression data. The relative prevalence of the two subclones associated with cell cycle and extracellular matrix pathways identified patients with significantly different survival outcomes. A radiogenomic signature-based predictive model significantly stratified patients into high- and low-risk groups with disparate disease-free survival (HR = 1.74, P = 0.003). Radiogenomic signatures were revealed as an independent predictive factor for CRC by multivariable analysis (HR = 1.59, 95% CI:1.03–2.45, P = 0.034). Functional analysis demonstrated that the 11 radiogenomic signatures were predominantly associated with extracellular matrix and immune-related pathways.

**Conclusions:**

The identified radiogenomic signatures might be a surrogate for genomic signatures and could complement the current prognostic strategies.

**Supplementary Information:**

The online version contains supplementary material available at 10.1186/s12967-022-03788-8.

## Background

Colorectal cancer (CRC) is the third leading cause of cancer-related deaths worldwide. Despite recent advancements in therapeutic techniques, the 5-year overall survival (OS) for this malignancy is only approximately 50% [1]. Therefore, there is an urgent need to develop prognostic biomarkers for improving CRC treatment. Substantial research has demonstrated that CRC is a heterogeneous disease with distinct molecular features and clinical responses [2–4]. An accurate understanding of the biological properties of CRC heterogeneity is essential for precise treatment, prediction of clinical prognosis, and the development of molecular subtype-specific targeted drugs.

Intratumour heterogeneity (ITH) is a hallmark of cancer that drives tumour evolution and disease progression. Increased ITH has been linked to a higher chance of recurrence, regardless of cancer type or treatment [5]. Therefore, exploration of ITH is helpful for the development of accurate prognostic tools. Previous studies have shown that the ITH of CRC can be characterised by massive parallel sequencing data [6–8]. Recent studies on CRC subtypes have employed unsupervised clustering to classify whole-genome expression profiles derived from bulk tumours. This unsupervised method has been effectively applied to a number of malignancies [9] but is less effective for mixtures with unknown compositions and noise. The deconvolution approach is an alternative unsupervised method that can estimate the underlying subclones of genomics in complex tissues to better understand tumour heterogeneity and predict prognosis [10].

Numerous studies on gene signature biomarkers have been published because of the advent of sequencing technology. However, their clinical applications are relatively limited. Current gene expression profiling methods are expensive, time-consuming, invasive, and require tumour biopsies for tissue extraction. Therefore, it was unavailable for all the patients. In contrast, radiomic biomarkers do not incur any additional expenses, because medical imaging is a routine part of the clinical decision-making process. Unlike biopsies, medical imaging is noninvasive and can provide information about the entire tumour phenotype, including ITH. Multiple studies have reported an association between radiomic characteristics and underlying gene expression patterns.

Radiogenomics explores the association between radiomic features and genomic characteristics, with the aim of revealing relevant features that reflect the underlying biological functions most related to clinical phenotypes. Numerous studies have established the viability of radiogenomics for identifying intrinsic molecular subtypes and gene expression profiles in cancers such as ovarian cancer [11], glioblastoma [12] and breast cancer [13]. Fan et al. [13] developed a radiogenomic signature to describe the landscape of breast cancer sub-clones and investigated their biological roles. Wu et al. [14] identified three imaging subtypes of breast cancer using dynamic MRI images and evaluated the prognostic value of these subtypes using public gene expression data.

**Fig. 1:**
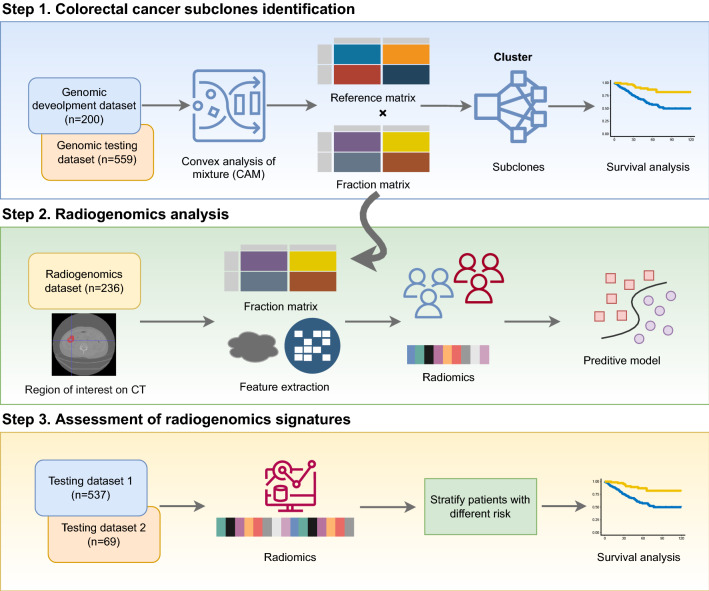
The workflow for integrative analysis of genomic and radiomic data. Step 1, unsupervised deconvolution method is employed to identify CRC subclones following by the biological function analysis. Step 2, prognostic predictive model is built based on radiogenomic signatures. Step 3, assessing the predictive model on imaging test datasets

**Fig. 2:**
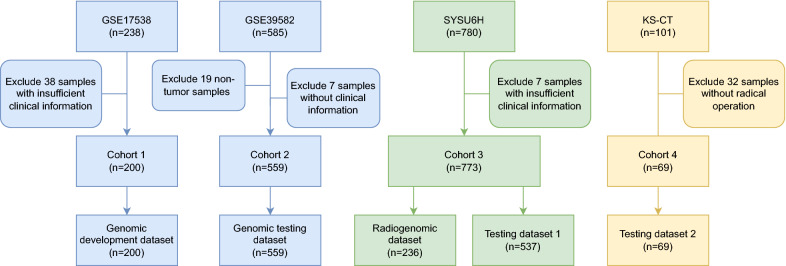
Data organization flowchart of four cohorts. A total of 1601 samples of colorectal cancer patients were included in five datasets of four cohorts according to the selection criteria

Here, we investigated the biological and clinical relevance of modelling multiscale ITH by conducting a radiogenomic analysis of 1601 samples from CRC patients on five datasets of four clinical cohorts (Figs. 1, 2).

## Methods

### Study design

Ethics approval for the retrospective review of imaging and clinical data was obtained from local ethics committees. The requirement for written informed consent was waived. The study was conducted in accordance with the most recent version of the Declaration of Helsinki. This study was conducted in three steps (Fig. 1). First, we identified four genomic subclones of CRC using unsupervised deconvolution analysis of publicly available genomic datasets. The biological functions of each subclone were explored using gene set enrichment analysis (GSEA) [15]. Based on the estimated proportion of each subclone, patients were divided into high- and low-risk groups by consensus clustering, and survival differences were compared between the groups. Second, we established radiogenomic signatures to link radiomic features with prognostic subclone compositions in an independent radiogenomic dataset containing matched imaging and gene expression data. Lastly, the prognostic value of the identified radiogenomic signatures was validated using two independent testing datasets containing imaging and survival information collected from different medical centres.

### Patient population and data collection

This multi-institutional retrospective study comprised five datasets: a genomic development dataset (n = 200), genomic testing dataset (n = 559), radiogenomic dataset (n = 236), and two image testing datasets (n = 543, 69). A total of 1601 patients diagnosed with CRC were enrolled (Fig. 2). Genomic development and testing datasets were retrieved from the Gene Expression Omnibus with accession numbers GSE17538 [16] and GSE39582 [8], respectively. CRC cohorts from the Sixth Affiliated Hospital of Sun Yat-sen University (SYSU6H) and the First People's Hospital of Kashi Prefecture (KSH) were used in the radiogenomic discovery and validation steps. The inclusion criteria were as follows: (a) pathologically confirmed CRC, (b) no history of neoadjuvant therapy, and (c) underwent contrast-enhanced CT within 30 days prior to surgery. Patients were further excluded if the CT images were of insufficient quality for analysis or lost to follow-up. The patients’ follow-up cutoff was November 2021. Matched CT scans and RNA sequencing data were available for 236 patients from SYSU6H (Radiogenomic dataset). Patients with only CT images from two independent medical centres were used as test datasets. In particular, patients with only CT images from SYSU6H were allocated to image testing dataset 1 and patients from KSH were allocated to image testing dataset 2. The demographic and clinical characteristics of patients from the two medical centres are summarised in Table 1. The methods were performed in accordance with the relevant guidelines and regulations, and were approved by SYSU6H.

**Table 1:** Demographic characteristics of patients

	Genomic development dataset	Genomic testing dataset	Radiogenomic dataset	Image testing dataset 1	Image testing dataset 2
n	200	559	236	537	69
Sex (%)					
F	98 (49)	251 (44.9)	101 (42.8)	225 (41.9)	39 (56.5)
M	102 (51)	308 (55.1)	135 (57.2)	312 (58.1)	30 (43.5)
Tumour grade (%)					
High	16 (8)	–	53 (22.5)	102 (19.0)	–
Low	29 (14.5)	–	17 (7.2)	30 (5.6)	57 (82.6)
Middle	155 (77.5)	–	135 (57.2)	167 (31.0)	12 (17.4)
Unknown	–	–	31 (13.1)	239 (44.4)	–
CEA (%)					
Abnormal	–	–	131 (55.5)	226 (41.6)	43 (62.3)
Normal	–	–	98 (41.5)	114 (21.0)	26 (37.7)
Unknown	–	–	7 (3.0)	203 (37.4)	–
TNM stage (%)					
I	28 (14)	32 (5.7)	22 (9.3)	84 (15.6)	–
II	70 (35)	262 (46.9)	65 (27.5)	188 (34.9)	12 (17.4)
III	75 (37.5)	202 (36.2)	64 (27.1)	187 (34.8)	41 (59.4)
IV	27 (13.5)	60 (10.7)	82 (34.7)	79 (14.7)	16 (23.2)
Unknown	–	3 (0.5)	3 (1.3)	–	–
DFS (months, mean (SD))	42.28(30.10)	48.74(40.38)	37.46 (26.70)	56.76 (35.17)	16.46 (4.26)
Event (%)					
Disease-free	145 (72.5)	379 (67.8)	157 (66.5)	349 (65.0)	41 (59.4)
Relapse	55 (27.5)	177 (31.7)	79 (33.5)	188 (35.0)	28 (40.6)
Unknown	–	3 (0.5)	–	–	–

All enhanced CT scans were acquired in the Digital Imaging and Communications in Medicine (DICOM) format. Experienced clinicians manually contoured the tumour regions of interest to arrive at a three-dimensional segmentation using ITK-snap (Version 3.2; http://itksnap.org/). All image processing and feature extraction processes were performed using the Pyradiomics package (21) on the Python platform (Version 2.7).

### Deconvolution analysis and modelling

The deconvolution method of convex analysis of the mixture [17] was employed to identify genomic subclones. The convex analysis of the mixture method postulates that the gene expression level is a linear combination of sub-population expression, and the weight contribution of a sub-population is proportional to its abundance and specific expression. The linear mixing model can be formulated as an ${\mathbf{X}} ={\mathbf{A}} \times {\mathbf{S}}$. Convex analysis of a mixture identifies molecular markers from the original mixed expression matrix(X) and generates a reference matrix(S) and a fraction matrix(A), where the reference matrix is a subclone-specific expression and the fraction matrix estimates the constituent proportion. Patients were stratified into high- and low-risk groups based on the fraction matrix using consensus clustering [18]. Then, the least absolute shrinkage and selection operator (LASSO) with Cox regression was applied to select radiogenomic signatures with nonzero coefficients. We performed a tenfold cross-validation procedure to optimise the parameters. A prognostic predictive model was built using an extreme learning machine [19] with 1000 hidden nodes. The trained model classified patients into high- and low-risk groups.

### Validation of the prognostic value of the radiogenomic signatures

We tested the prognostic capability of the radiogenomic signatures by assessing the association with disease-free survival (DFS) in two independent cohorts, including image testing dataset 1 from SYSU6H and testing dataset 2 from KSH.

### Statistical analysis

Statistical analyses were performed using the R software (version 3.6.2). Univariate and multivariate analyses were performed using the Cox proportional hazard regression model. LASSO logistics regression analysis was performed by the ‘glmnet’ R package. The Kaplan–Meier method was employed to estimate survival probability, and the log-rank test was used to determine survival differences. The optimal cut-point for continuous variables was determined by the cut-point function from the ‘survminer’ R package [20]. All analyses were considered statistically significant at a two-sided P value of < 0.05.

## Results

### The identification of CRC subclones

**Fig. 3:**
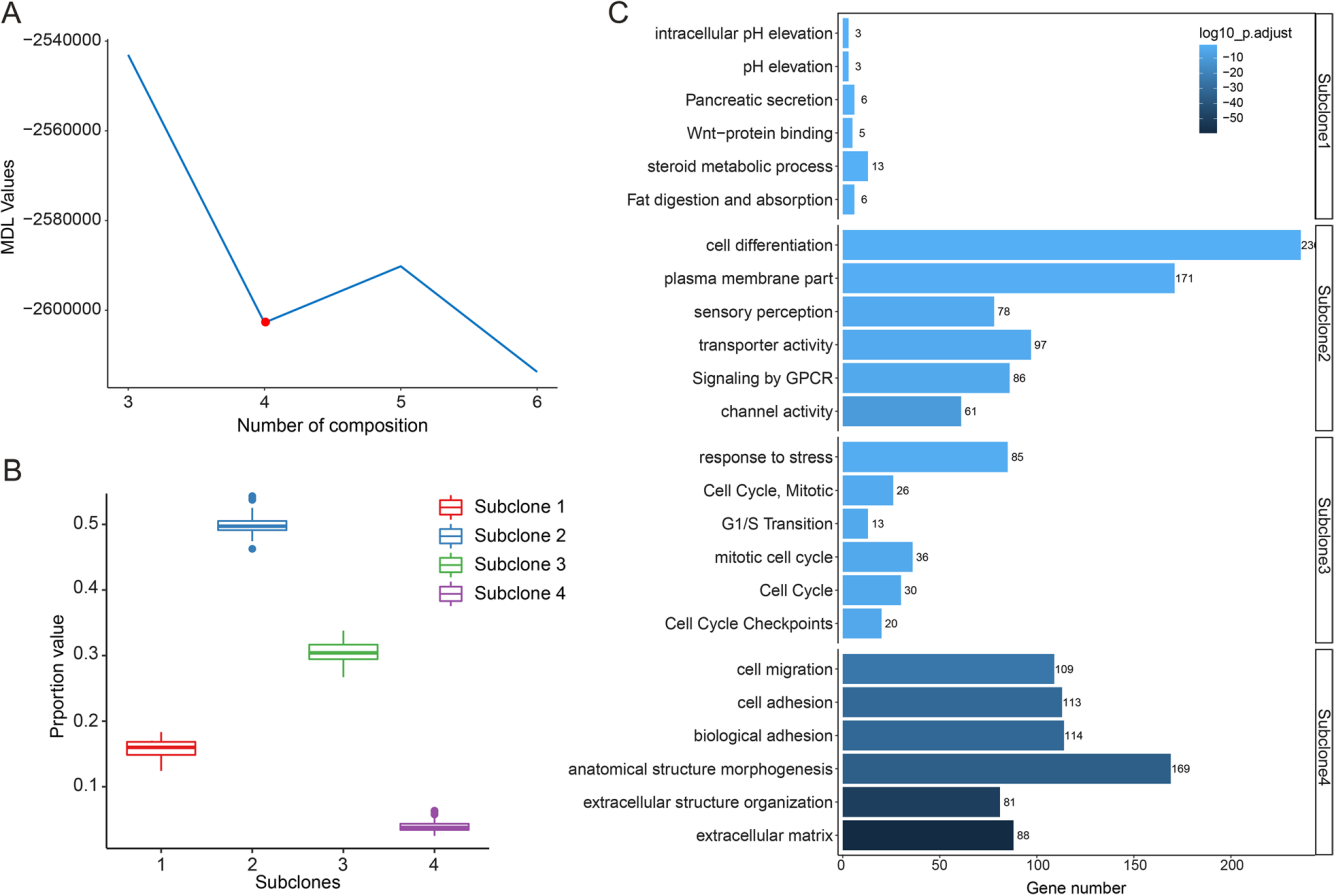
Four genomic subclones were identified by unsupervised deconvolution analysis. **A** Minimum description length curve determined the optimal clustering number k = 4. **B** Proportion distribution in each subclone. **C** GSEA analysis shown that the identified subclones are significantly enriched in cancer-related pathways

ITH was estimated at the genome level by unsupervised convex analysis of a mixture of gene expression data from GSE17538 (genomic development dataset, n = 200). The optimal number of subclones was determined using the minimum description length curve, a widely adopted information theoretical criterion. K = 4 was chosen based on the minimum description length, indicating there were four optimal CRC subclones (Fig. 3A). Among these four subclones, subclone 2 had the highest fraction proportion, accounting for approximately 49% of all subclones. The subclone with the lowest proportion was subclone 4, with only 3.56% (Fig. 3B). To investigate the biological roles of the identified subclones, GSEA was performed using subclone-specific marker genes inferred by convex analysis of the mixture method. GSEA analysis revealed that these four subclones are distinguished by distinct pathways (Fig. 3C): subclone 1 features the Wnt signalling pathway; subclone 2 is characterised by the cell differentiation pathway; subclone 3 is characterised by the cell cycle, which reflects cell proliferation and may be used to estimate prognosis [21]; subclone 4 is associated with the extracellular matrix (ECM), which regulates epithelial-to-mesenchymal transition, and dysregulated expression of related genes is associated with poor prognosis [22]. Based on the biological enrichment analysis, we found that subclone 1,3, and 4 recapitulated the previously reported CMS2, CMS1, and CMS4 of CRC consensus molecular subtypes (CMS) [3].

### Prognostic assessment of genomic subclones

**Fig. 4:**
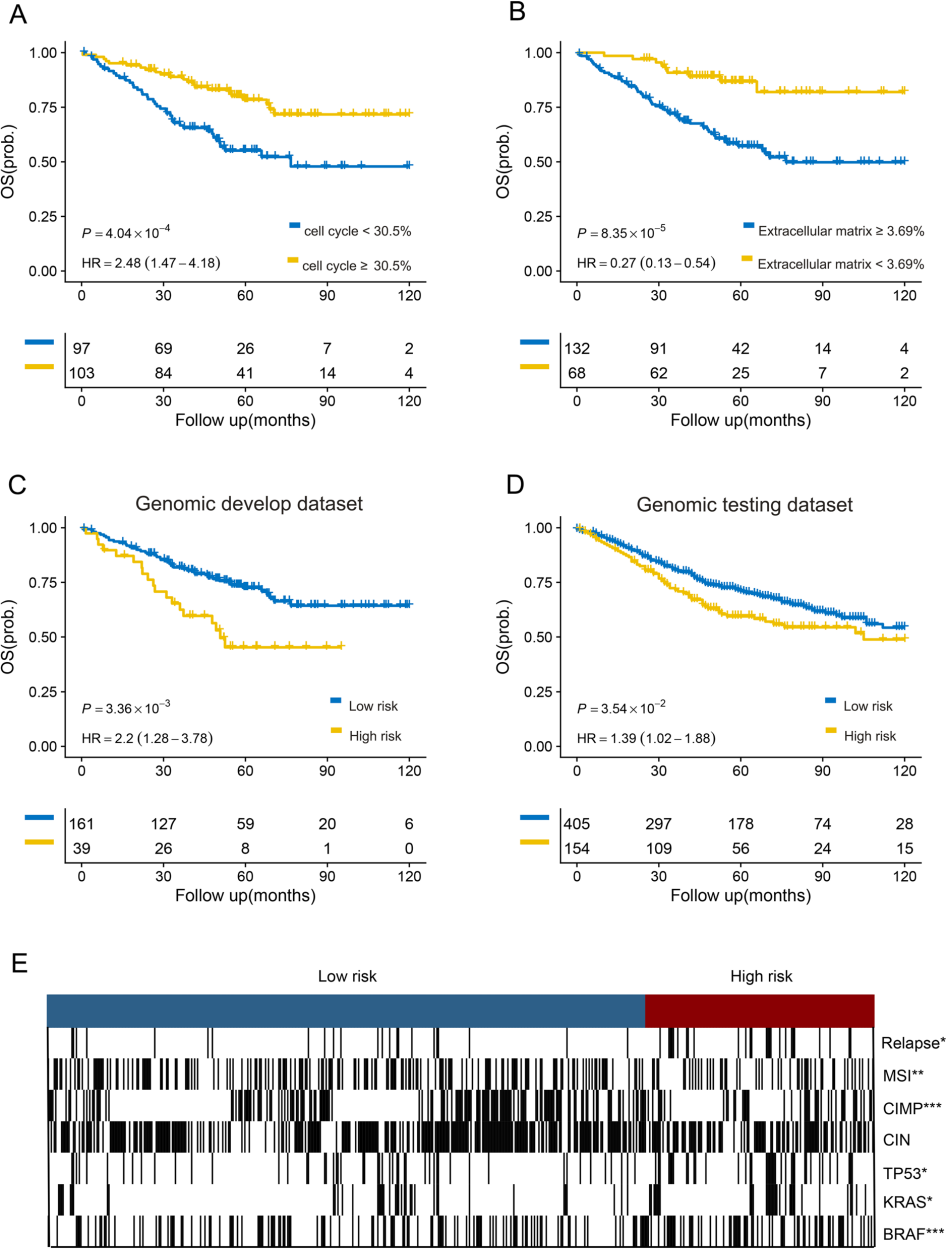
Genomic subclones correlate significantly with patient survival and clinicopathological factors. Kaplan–Meier curves show the significant association of proportion in cell cycle (**A**) and extracellular matrix (**B**) with overall survival. Genomic subclones stratified patients into high- and low-risk group with diversity in overall survival in the development dataset (**C**) and testing dataset (**D**). **E** The two patients’ groups show significant association with some clinicopathological factors (* < 0.05, ** < 0.01, *** < 0.001)

To examine the prognostic power of cancer-related pathways, including cell cycle and ECM subclones (subclones 3 and 4), we determined their correlation with OS in the development dataset (n = 200) by calculating the proportion of the total subclones. The cell cycle and ECM subclones stratified patients into poor and good OS groups, with significantly different survival rates (log-rank test P < 0.001, Fig. 4A; log-rank test P < 0.001, Fig. 4B). Briefly, tumours with a high proportion of cell cycle subclones exhibited good prognosis (Fig. 4A). In contrast, tumours containing the ECM subclone with a high prevalence were more likely to show inferior OS (Fig. 4B). To further investigate the prognostic capacity of these subclones, we applied consensus clustering based on the patient subclone composition. Patients were finally clustered into two groups, high-risk (n = 39) and low-risk (n = 161), based on cell cycle and ECM prognostic subclone compositions. There was a significant difference in the OS between the two groups (log-rank test, P = 0.003; HR, 2.2; Fig. 4C). To further evaluate the clinical relevance of the genomic subclones, we examined them using a second public CRC genomic dataset (GSE39582; n = 559). The fraction matrix of the genomic testing dataset was obtained on the basis of the reference matrix inferred from the genomic development dataset. Patients were also clustered into high- and low-risk groups according to the tumour subclone composition. Similarly, the high-risk group was associated with inferior survival (log-rank test, P = 0.025; HR, 1.4; Fig. 3D).

Using comprehensive data from the genomic testing dataset (GSE39582), we further investigated the correlation between the subclones and recognised CRC molecular biomarkers. The frequencies of various key mutations, such as TP53, KRAS, and BRAF, differed significantly across the subclone-clustered high- and low-risk groups (Fig. 4E). Microsatellite instability, BRAF, and CIMP + were enriched in the low-risk group. The increased incidence of TP53 and KRAS mutations in the high-risk group may lead to more aggressive tumours and poorer prognoses. Consistently, the high-risk group exhibited a significantly higher frequency of tumour recurrence (Fig. 4E). Taken together, the genomic subclones identified using unsupervised deconvolution analysis revealed a substantial correlation between ITH and patient prognosis.

### Radiogenomic signatures development

**Fig. 5:**
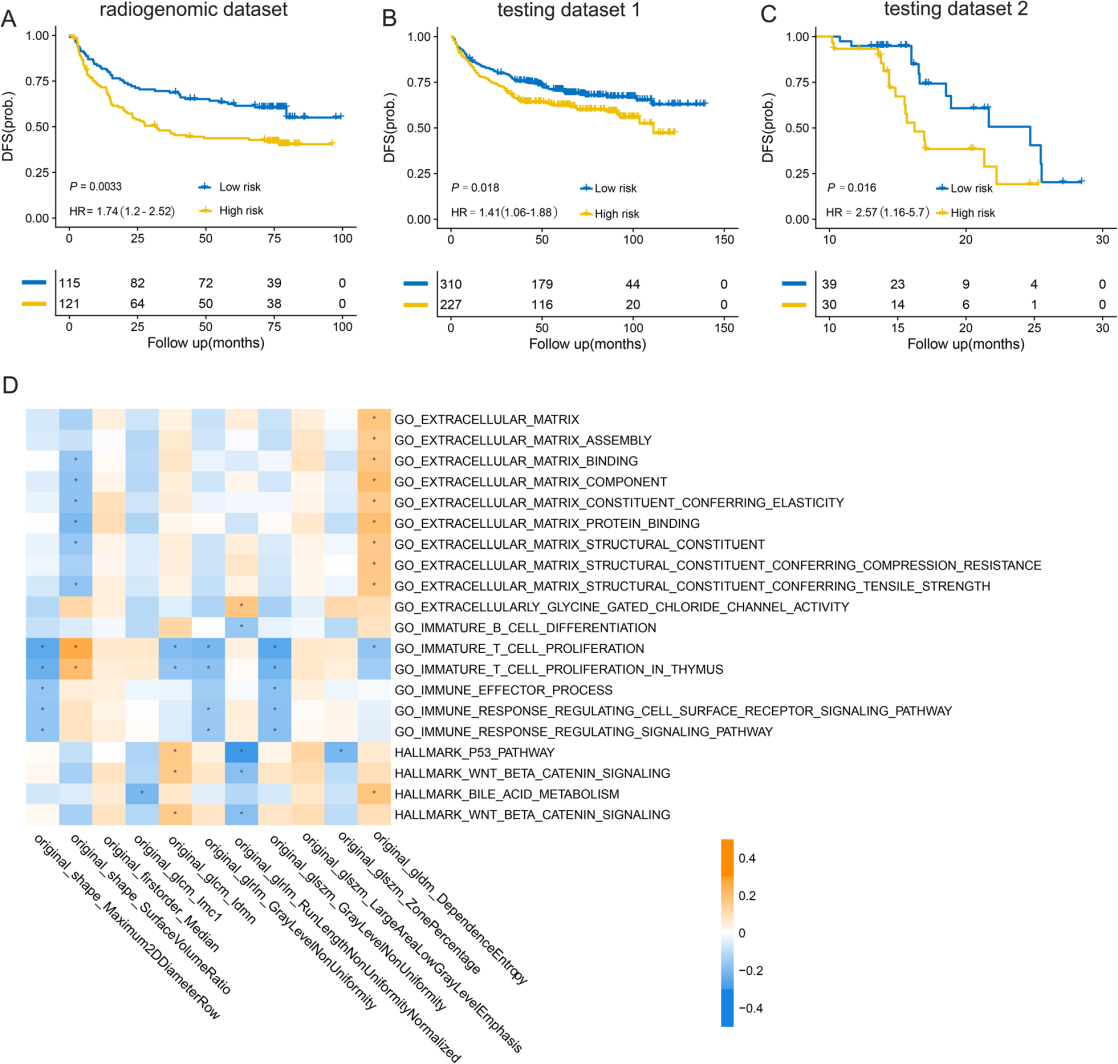
Radiogenomic signatures validated as predictors of colorectal cancer prognosis. Kaplan–Meier curves show the two groups developed on radiogenomic dataset (**A**), image testing dataset1 (**B**) and dataset2 (**C**) correlate significantly with patient disease-free survival. **D** The colour block in the heatmap represent the value of Pearson correlation coefficient and ‘*’ represents a statistically significant correlation (p < 0.05)

Radiogenomic signatures were established by linking radiomic features to genomic signatures of ITH. Patients from SYSU6H who contributed to the CT scans and gene expression profiles in the radiogenomic dataset (n = 236) were used to develop radiogenomic signatures. Under the supervision of the reference matrix inferred from the genomic development dataset, the gene expression profiles of the radiogenomic dataset were deconvoluted to obtain a fraction matrix. The patients were divided into high- and low-risk groups using consensus clustering and predictive subclone fractions. The two groups were strongly associated with DFS (log-rank test, P = 0.0033; Fig. 5A). Radiogenomic signatures were extracted from the enhanced CT imaging data and used to train a predictive model for classifying patients into high- and low-risk groups. We generated 100 radiomic features from the CT images. LASSO-Cox regression was performed on the radiogenomic dataset, and 11 features with nonzero coefficients were selected. Detailed information on the selected radiogenomic signatures is provided in Additional file 1: Table S1. Based on the clustered groups and selected radiogenomic signatures, we built a prognostic and predictive model using an extreme learning machine classifier with 1000 hidden nodes. This radiogenomic signature-based classifier has an accuracy of 0.97 in predicting each risk group.

### Prognostic assessment of radiogenomic signatures

The clinical significance of radiogenomic signatures was assessed by determining their ability to predict survival. Two image-testing datasets (n = 543; n = 69) were used to evaluate the clinical relevance of the radiogenomic signatures. The radiogenomic signatures successfully distinguished patients into high- and low-risk groups with diverse DFS (test dataset 1: P = 0.018; Fig. 5B; test dataset 2: P = 0.016; Fig. 5C), demonstrating their efficacy as prognostic predictors of CRC.

To examine whether the proposed radiogenomic signatures were independent prognostic factors, we performed multivariate Cox regression analyses on both the radiogenomic and testing datasets. Available clinical variables applied for analysis included clinicopathological factors such as sex (male vs. female), carcinoembryonic antigen (CEA, normal < 5 ng/ml vs. abnormal >  = 5 ng/ml), tumour grade (low vs. middle and high), and TNM stage (I-II vs. III-IV). The serum CEA level and TNM stage were identified as prognostic risk factors in the radiogenomic dataset (P = 0.001 and P < 0.001, respectively) and imaging test dataset 1 (P = 0.025 and P < 0.001, respectively); however, their significance was lost in image testing dataset 2. Radiogenomic signatures were the only independent prognostic factors for DFS across all three datasets (P = 0.03, P = 0.05, and P = 0.01, respectively) (Table 2).

**Table 2:** Multivariable analysis of the prognostic value of radiogenomic signatures and other clinical factors

	Radiogenomics dataset		Imaging test dataset 1		Imaging test dataset 2	
	HR (95% CI)	*P*	HR (95% CI)	*P*	HR (95% CI)	*P*
Sex:(M vs. F)	1.33 (0.87–2.05)	0.17	1.48 (1.03–2.14)	0.03	1.14 (0.5–2.65)	0.75
CEA:(Abnormal vs. Normal)	1.95 (1.29–2.96)	0.0014	1.51 (1.5–2.19)	0.025	1 (0.42–2.37)	0.98
Tumour grade: (Low vs. Middle + High)	1.22 (0.66–2.25)	0.52	1.94 (0.94–4)	0.07	1.74 (0.72–4.23)	0.22
TNM stage: (I + II vs. III + IV)	4.29 (2.5–7.38)	< 0.001	2.74 (1.84–4.1)	< 0.001	2.24 (0.81–6.2)	0.12
Radiogenomics groups: (High risk vs. Low risk)	1.59 (1.03–2.45)	0.034	1.42 (1–2.02)	0.05	2.78 (1.21–6.34)	0.01

### Biological function analysis of the radiogenomic signatures

The aforementioned findings indicate that radiogenomic signatures can serve as noninvasive surrogates for genomic signatures. To investigate the biological processes linked to the radiogenomic signatures, we ran GSEA with a gene expression profile for each patient based on the Molecular Signatures Database [15]. The gene expression of 236 patients from the radiogenomic dataset was used to calculate the enrichment score using the DeepCC tool [23]. Spearman’s correlations were calculated between the 11 radiogenomic signatures and the enrichment scores for specific dysregulated molecular pathways. The 11 radiogenomic signatures were strongly related to the ECM and immune-related pathways. The original_gldm_DependenceEntropy was significantly enriched in the extracellular matrix pathways, whereas the original_shape_SurfaceVolumeRatio was significantly enriched in the immune pathways (Fig. 5D). The value of the original_shape_SurfaceVolumeRatio is the ratio of the surface area to the volume and partly depends on the volume of the tumour region. We found that the patients’ relapse probability increased with decreasing surface volume ratio value (t-test, P = 0.02, Additional file 1: Figure S1).

## Discussion

In this multi-institutional study, we conducted a radiogenomic analysis of 1601 CRC samples from five datasets to investigate ITH and establish radiogenomic signatures to predict prognosis. Integrative analysis employing radiomics and genomics shows great promise in unravelling ITH and predicting CRC prognosis.

ITH is gaining recognition as a factor in aggressive disease development and resistance to therapy. ITH is a negative prognostic factor in patients with various solid malignancies [24]. In addition, a comprehensive study showed that the mesenchymal features of tumours are highly correlated with ITH and immunosuppressive pathways [25]. Numerous studies have sought to investigate ITH by comprehensively analysing genomic data [26, 27]. However, the clinical utility of ITH is limited. Although high-throughput sequencing can provide a great deal of biological information, it is limited by expense, invasion, and the potential for sampling bias caused by ITH. Imaging, on the other hand, provides a unique opportunity for non-invasive interrogation of the entire tumour and its surrounding tissues, and may provide crucial supplementary information for molecular research. Non-invasive imaging markers derived from routine clinical images have been increasingly researched to provide insights into the tumour microenvironment. Radiogenomics, on the other hand, integrates image characteristics with genomic features, harnesses the noninvasive benefits of radiomics, and simultaneously leverages the power of genomics to dramatically improve the interpretability of models.

We built a prognosis prediction model based on radiogenomic signatures that capture the underlying relationships between prognostic genomic signatures and radiomic signatures of ITH. An unsupervised deconvolution approach was performed on the gene expression profiles of CRC primary tumours to dissect ITH and identify the four genomic subclones. Different subclone compositions reflect ITH in tumours and are predictive of patient survival. Prognostic-relevant genomic signatures of CRC were subsequently generated from the proportional compositions of key predictive subclones. By mapping these genomic signatures to radiomic signatures, radiogenomic signatures were created. Finally, a clinically useful predictive model was constructed using the radiogenomic signatures and survival data.

Our research showed that radiogenomic signatures may be a suitable substitute for genomic signatures. Even with imaging-only data input, validation results of test cohorts from two separate medical centres indicated that our radiogenomic prognostic prediction model could effectively stratify the prognostic risk of CRC patients (Fig. 5B, C). A robust predictive model was constructed by establishing a link between genomics and radiomics. In the process of applying the model, only image data is required in the absence of genomic data, which dramatically lowers the threshold for clinical application of the model. Currently, imaging examinations are routinely used for tumour diagnosis and therapy decisions. Utilising images as input data for prognostic prediction models does not significantly increase healthcare expenditure. Furthermore, imaging examinations are noninvasive and can be repeated at various times. CT-based radiogenomic signatures allow us to forecast patient prognosis and ITH prior to surgery.

Owing to the construction of a link between genomics and radiomics, the model is substantially more interpretable. Imaging characteristics have been related to CRC outcomes, such as treatment response, lymph node metastasis, local recurrence, and survival [28–30], but their biological underpinnings remain unclear. In the present study, we did not introduce relevant prior information but identified four CRC genomic subclones by analysing a large number of gene expression profiles using a fully unsupervised deconvolution strategy. According to our study, tumours with a low proportion of cell cycle subclones and a high proportion of extracellular matrix subclones were associated with a shorter survival rate. Among the signalling pathways within the cell cycle subclone, the G1/S transition and cell cycle checkpoint pathways likely reflect the DNA damage response and can be exploited for prognosis [31]. Cell cycle checkpoints can repair DNA and prevent further damage by detecting damaged DNA and temporarily halting the cell cycle progression. Cell cycle dysregulation can lead to abnormal cell proliferation and apoptosis, and is responsible for tumourigenesis. Defects in cell cycle checkpoints may be a cause of genomic instability in tumours [32]. Therefore, abnormalities in cell cycle pathways have prognostic significance in CRC. The ECM subclone is another subclone strongly associated with prognosis. It is reported that ECM remodelling is associated with CRC carcinogenesis and progression [33, 34]. As a major component of the tumour microenvironment, the ECM plays a crucial role in tumour progression and treatment response. Chakravarthy et al. built a signature that linked extracellular matrix genes to immune evasion and immunotherapy failure [35]. Eleven radiomic characteristics were chosen for our model, the majority of which were enriched in ECM- and immune-related pathways, which are well-known prognosis-related pathways. This suggests that the prognostic value of these radiomic signatures has a biological foundation. These morphological textures and spatial features are inseparable from the gene- and cell-level characteristics. Machine learning helps us better understand the biology behind these morphological textures and spatial features. Using this three-step methodology, we created a prognostic prediction model that provides an entry point for elucidating the underlying molecular mechanisms.

Our study had several limitations. First, this was a retrospective study, which led to inevitable disadvantages. In follow-up research, these findings should be validated by prospective studies to reduce the bias caused by uncontrollable factors in retrospective studies. Second, our genomic development dataset and corresponding testing dataset were obtained from public databases. However, cohorts 3 and 4 came from local medical centres and provided in-house data. CT scans from different machines at different centres better validate the robustness and clinical usability of the model. Third, the regions of interest are manually annotated, and this process is time-consuming and tedious. We are currently investigating more robust semi-automatic annotation methods [36] to address this issue.

## Conclusions

In conclusion, we conducted an integrative analysis of genomics and radiomics to dissect ITH and build models for predicting the prognosis of patients with CRC. The unsupervised deconvolution method for genomic subclone identification provides a new perspective for exploring tumour heterogeneity. Radiogenomic signatures can be independent prognostic biomarkers and may serve as surrogates for genomic signatures. This integrative analysis of the radiogenomic strategy shows great promise for understanding ITH, and can be extended to other cancers to help patients who might benefit from precise clinical treatment.

## Supplementary Information

**Additional file 1: Figure S1.** Radiomic features used to predict the prognosis of the CRC risk groups. (A)Example of patients in the low risk with radiomics feature (SurfaceVolmeRation) value of 0.82 and in the high-risk group with value of 0.29. The regions of interest (ROI) for tumour(red) are shown. (B) Boxplot of SurfaceVolmeRation value within radiogenomics dataset for the low risk and high risk radiomics groups. **Table S1.** Selected imaging feature description.

## Data Availability

The datasets used and/or analysed during the current study are available from the corresponding author on reasonable request.

## Abbreviations

CRC: Colorectal cancer
ITH: Intratumor heterogeneity
OS: Overall survival
GSEA: Gene set enrichment analysis
SYSU6H: The Sixth Affiliated Hospital of Sun Yat-sen University
KSH: The First People's Hospital of Kashi Prefecture
DFS: Disease-free survival
ECM: Extracellular Matrix
