# Supplementary material for: CT-based radiogenomic analysis dissects intratumor heterogeneity and predicts prognosis of colorectal cancer: a multi-institutional retrospective study
Source: J Transl Med. 2022 Dec 8;20:574. doi: 10.1186/s12967-022-03788-8 (PMC9730572; doi:10.1186/s12967-022-03788-8)
Supplement: Supplementary file 1 — Additional file 1: Figure S1. Radiomic features used to predict the prognosis of the CRC risk groups. (A)Example of patients in the low risk with radiomics feature (SurfaceVolmeRation) value of 0.82 and in the high-risk group with value of 0.29. The regions of interest (ROI) for tumour(red) are shown. (B) Boxplot of SurfaceVolmeRation value within radiogenomics dataset for the low risk and high risk radiomics groups. Table S1. Selected imaging feature description. [file 12967_2022_3788_MOESM1_ESM.docx]

**Supplementary Table 1. Selected imaging feature description**

| Feature class | Feature description |
| --- | --- |
| First Order Features | Median |
| Shape Features (3D) | Maximum2DDiameterRow, SurfaceVolumeRatio |
| Gray Level Co-occurrence Matrix (GLCM) Features | Informational Measure of Correlation (IMC) 1, Inverse Difference Moment Normalized (IDMN) |
| Gray Level Run Length Matrix (GLRLM) Features | GrayLevelNonUniformity, RunLengthNonUniformityNormalized |
| Gray Level Size Zone Matrix (GLSZM) Features | GrayLevelNonUniformity, LargeAreaLowGrayLevelEmphasis, ZonePercentage |
| Gray Level Dependence Matrix (GLDM) Features | DependenceEntropy |


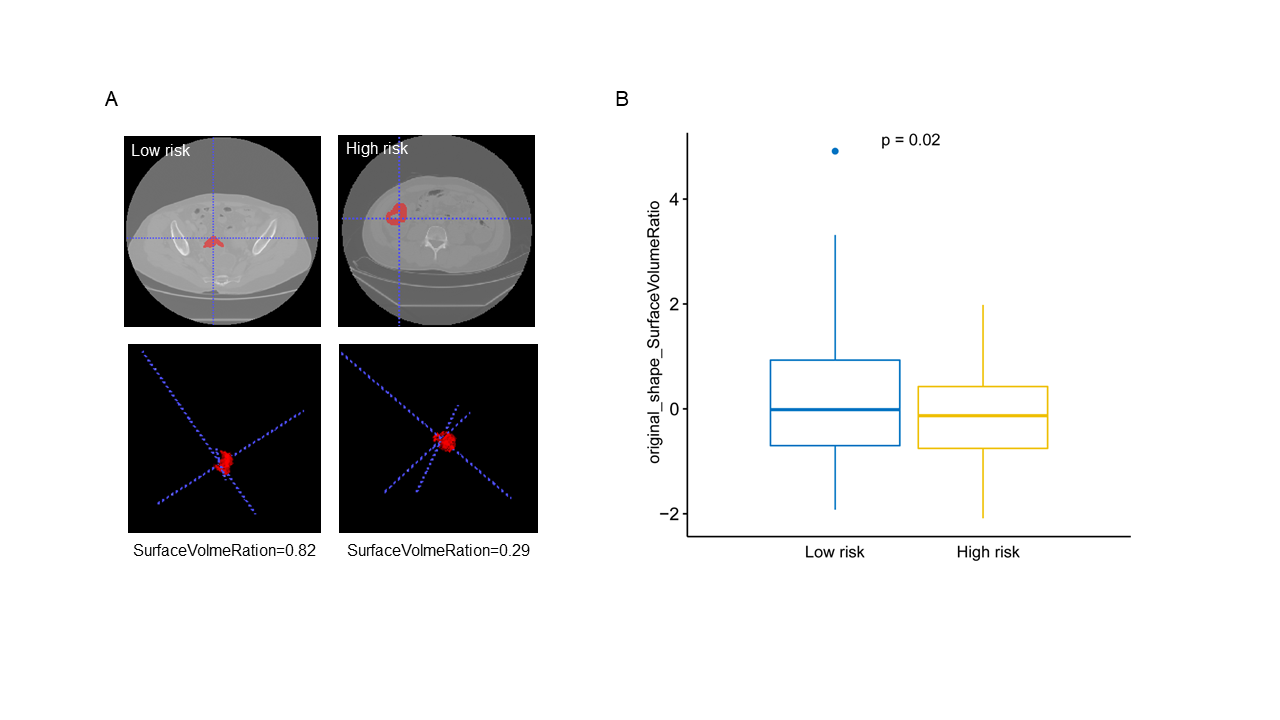


**Figure S1. Radiomic features used to predict the prognosis of the CRC risk groups.** (A)Example of patients in the low risk with radiomics feature (SurfaceVolmeRation) value of 0.82 and in the high-risk group with value of 0.29. The regions of interest (ROI) for tumor(red) are shown. (B) Boxplot of SurfaceVolmeRation value within radiogenomics dataset for the low risk and high risk radiomics groups.
